# Association of tamoxifen use and reduced risk of contralateral breast cancer for BRCA1 and BRCA2 mutation carriers

**DOI:** 10.1186/1897-4287-10-S2-A11

**Published:** 2012-04-12

**Authors:** KA Phillips, RL Milne, MA Rookus, D Goldgar, M Friedlander, SA McLachlan, S Buys, AC Antoniou, K Birch, MB Terry, DF Easton, P Weideman, M Daly, N Andrieu, EM John, MJ Hooning, IL Andrulis, T Caldes, H Olsson, JL Hopper

**Affiliations:** 1 Peter MacCallum Cancer Centre, Melbourne, Australia; 2 University of Melbourne, Melbourne, Australia; 3 Netherlands Cancer Institute, Amsterdam, Netherlands; 4 University of Utah, Salt Lake City, USA; 5 Prince of Wales Hospital, Randwick, Australia; 6 St Vincent’s Hospital, Melbourne, Australia; 7 Huntsman Cancer Institute, Salt Lake City, USA; 8 University of Cambridge, Cambridge, UK; 9 Columbia University, New York, USA; 10 Fox Chase Cancer Center, Philadelphia, USA; 11 Institut Curie, Paris, France; 12 Northern California Cancer Center, Fremont, USA; 13 Erasmus University Medical Center Daniel den Hoed Cancer Center, Rotterdam, Netherlands; 14 Ontario Cancer Genetics Network, Cancer Care Ontario, Toronto, Canada; 15 Hospital Clinico San Carlos, Madrid, Spain; 16 Lund University Hospital, Lund, Sweden

## Background

The efficacy of tamoxifen as a breast cancer (BC) prevention strategy for *BRCA1* and *BRCA2* mutation carriers is uncertain.

## Patients and methods

Female *BRCA1* and *BRCA2* mutation carriers, with a personal history of BC since 1970, enrolled in any of the BC family studies, kConFab (Kathleen Cuningham Foundation Consortium for Research into Familial Breast Cancer), IBCCS (International BRCA1 and BRCA2 Carrier Cohort Study), or BCFR (Breast Cancer Family Registry) were eligible. Those with bilateral disease at first BC diagnosis, tamoxifen use prior to their first BC diagnosis, or another invasive cancer with were excluded. Data were self-reported at entry into the cohort and at follow-up. Hazard ratios (HRs) for development of contralateral BC associated with tamoxifen use after first BC diagnosis were estimated using Cox proportional hazards, modeling time from diagnosis of first primary BC, adjusting for year of birth (continuous), age at diagnosis (continuous), country and bilateral oophorectomy (yes/no, time-varying). Data were censored at contralateral mastectomy, death or loss to follow-up.

## Results

Of 1642 *BRCA1* and 919 *BRCA2* mutation carriers, 374 (23%) and 444 (48%), respectively, took tamoxifen after their first BC diagnosis. During 21,344 person-years of follow-up, 596 contralateral BCs were observed. Overall, the adjusted HR estimates were 0.31 (95% CI: 0.22-0.45) and 0.24 (95% CI 0.16-0.35) for *BRCA1* and *BRCA2* mutation carriers, respectively. After left-truncating the analysis at time of recruitment, the adjusted HR estimates were 0.52 (95% CI: 0.26-1.04) and 0.39 (95% CI: 0.17-0.89) from studying 629 *BRCA1* and 412 *BRCA2* mutation carriers, respectively, with 4,869 person-years of follow-up.

## Conclusions

Although biased estimates due to non-random use of tamoxifen cannot be excluded, these results are consistent with tamoxifen reducing BC risk for both *BRCA1* and *BRCA2* mutation carriers.

